# Prevalence of antimicrobial resistance among bacterial pathogens isolated from cattle in different European countries: 2002–2004

**DOI:** 10.1186/1751-0147-50-28

**Published:** 2008-07-08

**Authors:** Rene S Hendriksen, Dik J Mevius, Andreas Schroeter, Christopher Teale, Danièle Meunier, Patrick Butaye, Alessia Franco, Andra Utinane, Alice Amado, Miguel Moreno, Christina Greko, Katharina Stärk, Christian Berghold, Anna-Liisa Myllyniemi, Dariusz Wasyl, Marianne Sunde, Frank M Aarestrup

**Affiliations:** 1 National Food Institute, Technical University of Denmark, Bülowsvej 27, DK-1790, Copenhagen V, Denmark; 2 Central Institute for Animal Disease Control, PO Box 2004, Edelhertweg 15, 8203, Lelystad, The Netherlands; 3 Federal Institute for Risk Assessment, Postfach 330013, Diedersdorferweg 1, 14191, Berlin, Germany; 4 Veterinary Laboratories Agency, Kendal Road, Harlescott, SY1 4HD, Shrewsbury, UK; 5 Agence Française de Sécurité Sanitaire des Aliments, Lyon LERPBV, 31 avenue Tony Garnier, 69000, Lyon, Cedex 07, France; 6 Veterinary and Agrochemical Research Centre, Groeselenberg 99, B-1180, Ukkel, Belgium; 7 Istituto Zooprofilattico Sperimentale delle Regioni Lazio e Toscana, Via Appia Nuova 1411, 00178 Roma, Italy; 8 State Veterinary Medicine Diagnostic Centre of Food and Veterinary Service, Lejupes 3, 1076, Riga, Latvia; 9 Laboratorio National de Investigacáo Veterinaria, Estrada de Benfica 701, 1549-011, Lisboa, Portugal; 10 Complutense University of Madrid, Ciudad Universitaria, 28040, Madrid, Spain; 11 National Veterinary Institute, Travv. 20, 75189, Uppsala, Sweden; 12 Bundesamt für Veterinärwesen, Schwarzenburgstrasse 161, 3003, Bern, Switzerland; 13 Agency for Health and Food Safety, Beethovenstrasse 6, A-8010, Graz, Austria; 14 Finnish Food Safety Authority, PO Box 45, Hameentie 57, 00581, Helsinki, Finland; 15 National Veterinary Research Institute, Partyzantow 57, 24-100, Pulawy, Poland; 16 National Veterinary Institute, PO Box 8156 Dep, Ullevaalsveien 68, 0033, Oslo, Norway

## Abstract

**Background:**

The project "Antibiotic resistance in bacteria of animal origin – II" (ARBAO-II) was funded by the European Union (FAIR5-QLK2-2002-01146) for the period 2003–2005, with the aim to establish a continuous monitoring of antimicrobial susceptibility among veterinary laboratories in European countries based on validated and harmonised methodologies. Available summary data of the susceptibility testing of the bacterial pathogens from the different laboratories were collected.

**Method:**

Antimicrobial susceptibility data for several bovine pathogens were obtained over a three year period (2002–2004). Each year the participating laboratories were requested to fill in excel-file templates with national summary data on the occurrence of antimicrobial resistance from different bacterial species.

A proficiency test (EQAS – external quality assurance system) for antimicrobial susceptibility testing was conducted each year to test the accuracy of antimicrobial susceptibility testing in the participating laboratories. The data from this testing demonstrated that for the species included in the EQAS the results are comparable between countries.

**Results:**

Data from 25,241 isolates were collected from 13 European countries. For *Staphylococcus aureus *from bovine mastitis major differences were apparent in the occurrence of resistance between countries and between the different antimicrobial agents tested. The highest frequency of resistance was observed for penicillin. For *Mannheimia haemolytica *resistance to ampicillin, tetracycline and trimethoprim/sulphonamide were observed in France, the Netherlands and Portugal. All isolates of *Pasteurella multocida *isolated in Finland and most of those from Denmark, England (and Wales), Italy and Sweden were susceptible to the majority of the antimicrobials. *Streptococcus dysgalactiae *and *Streptococcus uberis *isolates from Sweden were fully susceptible. For the other countries some resistance was observed to tetracycline, gentamicin and erythromycin. More resistance and variation of the resistance levels between countries were observed for *Escherichia coli *compared to the other bacterial species investigated.

**Conclusion:**

In general, isolates from Denmark, England (and Wales), the Netherlands, Norway, Sweden and Switzerland showed low frequencies of resistance, whereas many isolates from Belgium, France, Italy, Latvia and Spain were resistant to most antimicrobials tested. In the future, data on the prevalence of resistance should be used to develop guidelines for appropriate antimicrobial use in veterinary medicine.

## Background

Antimicrobial agents are widely used for the treatment of bovine mastitis, respiratory tract infections and diarrhoea in cattle. During acute infections and outbreaks of infectious disease in groups or herds it is important to use an effective antimicrobial treatment as early as possible. This empirical treatment is generally based on knowledge of the resistance pattern of the different bacterial pathogens to antimicrobial agents used in the particular animal species.

Antimicrobial resistance is an increasingly important problem among several bacterial species causing infection in animals and humans in recent year. The problem for some bacterial species is so critical that there is few treatment options left [1,2].

The initial treatment of animals is commonly based on the experience regarding the expected resistance of the infectious agent. The fact that occurrence of antimicrobial resistance varies between countries and regions has the potential to complicate that matter. Furthermore, knowledge of expected resistance is limited by the small proportion of different bacterial pathogens from infected animals that actually are investigated for their antimicrobial resistance pattern.

To address this the ARBAO-II-project was funded by the European Union (FAIR5-QLK2-2002-01146) for the period 2003–2005, The aim of this project was to establish a continuous monitoring of antimicrobial susceptibility among veterinary laboratories in European countries based on validated and harmonised methodologies. Available summary data of the susceptibility testing of the bacterial pathogens from the different laboratories were collected and is herewith made publicly available.

In this report the first data on the prevalence of antimicrobial resistance among bacterial pathogens isolated from cattle are reported.

## Methods

### Participating laboratories

Each year the laboratories participating in the project were requested to fill in excel-file templates with national summary data on the occurrence of antimicrobial resistance from different bacterial species and groups. The participants were asked to submit national data but if this was not possible they were advised to submit regional or institutional data as done by England (and Wales). The data for some countries might be incomplete e.g. Latvia, Finland and Portugal and data were deducted for others if the number of isolates were lower than 9. The bacterial species and their antimicrobial resistances are given in Table 1 and Tables 2, 3, 4, 5, 6, respectively.

**Table 1:** Data of antimicrobial susceptibility submitted from the participating laboratories in the different European countries during a three years period.

Bacterial species	Year	Country and number of bacterial species													Total No. of isolates
		
		B	D	E^c^	FIN	F^a^	I	LV	NL	P	N	ES	S	CH	
*S. aureus*	2002	-	101	688	-	23–174	-	-	110	-	-	147	100	-	1 170–1 321
	2003	-	99	378	-	8–233	63	-	107	-	117	192	-	60	1 024–1 249
	2004	-	-	340	-	-	-	59	99	-	-	112	-	81	691
*M. haemolytica*	2002	-	-	78	-	20–56	-	-	-	57	-	-	-	-	155–191
	2003	-	19	90	-	4–68	9	-	35	-	-	-	-	-	157–221
	2004	-	-	101	-	-	-	-	16	-	-	-	-	-	117
*P. multocida*	'2002	-	26	113	-	21–85	-	-	-	-	-	-	-	-	160–224
	2003	-	48	154	15	10–218	24	-	45	-	-	-	-	-	296–504
	2004	-	-	178	-	-	-	-	16	-	-	-	-	-	194
*S. dysgalactiae*	2002	-	-	278	-	27–36	-	-	107	-	-	-	100	-	512–521
	2003	-	-	193	-	41–51	-	-	94	-	-	-	-	-	328–338
	2004	-	-	165	-	-	-	-	90	-	-	-	-	-	255
*S. uberis*	2002	-	-	1 195	-	195–291	-	-	103	-	-	-	98	-	1 591–1 687
	2003	-	-	775	-	471–610	27	-	83	-	-	-	-	-	1 356–1 495
	2004	-	-	885	-	-	-	-	99	-	-	-	-	-	984
*E. coli *(infections)	2002	102^b^	101	4 193	-	49–1 234	50	-	105	-	-	136	-	-	4 736–5 921
	2003	56^b^	57	2 659	-	150–1 835	75	-	153	-	-	155	169	16	3 490–5 175
	2004	237^b^	79	3 576	-	-	28		101	-	-	87	29	16	4153

Total	2002	102	228	6 545	0	335–1 876	50	0	425	57	0	283	298	0	
	2003	56	223	4 249	15	684–3 015	198	0	517	0	117	347	169	76	
	2004	237	79	5 245	0	0	28	59	421	0	0	199	29	97	

^a^: Multicenter study. The isolates were tested for different panels of antimicrobial agents in the different centers. ^b^: Veal calves. ^c^: Isolates from England included also Wales and have been collected by The Veterinary Laboratory Agency. The remaining part of the United Kingdom has separate laboratories. B – Belgium; DK – Denmark; E – England, FIN – Finland; F – France; I – Italy; LV – Latvia; NL – The Netherlands; P – Portugal; N – Norway; ES – Spain; S – Sweden; CH – Switzerland.

**Table 2:** Occurrence of antimicrobial resistance among *Staphylococcus aureus *from bovine mastitis in different European countries.

Antimicrobial agent	Year	Country and prevalence of resistance									
		
		DK	E	F	I	LV	NL	N	ES	S	CH
Chloramphenicol	2002	0.0	-	-	-	-	-	-	0.5	1.0	-
	2003	0.0	-	0.0	1.0	-	-	0.0	0.0	-	-
	2004	-	-	-	-	2.1	-	-	1.8	-	-
Erythromycin	2002	1.0	3.0	11.4	-	-	0.9	-	2.0	1.0	-
	2003	0.0	4.0	7.0	4.0	-	0.0	1.0	4.2	-	0.0
	2004	-	2.0	-	-	0.0	0.0	-	1.8	-	3.7
Gentamicin	2002	0.0	-	2.2	-	-	-	-	0.0	0.0	-
	2003	0.0	-	1.0	1.0	-	-	0.0	-	-	30
	2004	-	-	-	-	3.1	-	-	-	-	2.5
Oxacillin	2002	0.0	-	8.3	-	-	0.0	-	0.5	0.0	-
	2003	-	-	1.0	0.0	-	0.0	0.0	0.0	-	-
	2004	-	-	-	-	0.0	0.0	-	3.7	-	0.0
Penicillin	2002	29.7	46.0	4.7	-	-	10.0	-	45.0	7.0	-
	2003	23.1	36.0	3.0	43.0	-	24.3	5.0	40.1	-	-
	2004	-	38.0	-	-	49.0	12.1	-	33.0		17.3
Streptomycin	2002	4.0	-	7.5	-	-	1.8	-	-	1.0	-
	2003	1.0	-	8.0	8.0	-	1.9	2.0	6.3	-	5.0
	2004	-	-	-	-	0.0	2.0	-	6.3	-	-
Sulphonamides	2002	4.0	-	59.2	-	-	-	-	0.0	-	-
	2003	0.0	-	2.0	0.0	-	-	-	-	-	0.0
	2004	-	-	-	-	-	-	-	0.0	-	-
Tetracycline	2002	3.0	8.0	9.2	-	-	0.0	-	3.5	0.0	-
	2003	2.0	3.0	9.0	13.0	-	2.8	0.0	2.1	-	-
	2004	-	4.0	-	-	2.1	2.0	-	5.4	-	6.2
TMP + Sulphonamides	2002	-	-	1.7	-	-	0.0	-	-	0.0	-
	2003	-	-	0.0	0.0	-	-	-	-	-	-
	2004	-	-	-	-	0.0	0.0	-	-	-	-
Trimethoprim	2002	1.0	-	11.1	-	-	-	-	4.5	-	-
	2003	1.0	-	0.0	-	-	-	0.0	3.1	-	0.0
	2004	-	-	-	-	2.9	-	-	0.0	-	-
Neomycin	2002	0.0	0.4	-	-	-	-	-	-	-	-
	2003	-	0.0	2.0	-	-	-	1.0	-	-	0.0
	2004	-	0.3	-	-	5.3	0.0	-	-	-	-
Ceftiofur	2002	0.0	-	4.2	-	-	-	-	0.0	-	-
	2003	0.0	-	1.0	-	-	-	-	0.0	-	-
	2004	-	-	-	-	0.0	-	-	0.9	-	-

DK – Denmark; E – England, F – France; I – Italy; LV – Latvia; NL – The Netherlands; P – Portugal; N – Norway; ES – Spain; S – Sweden; CH – Switzerland.

**Table 3:** Occurrence of antimicrobial resistance among *Mannheimia haemolytica *isolated from cattle in different European countries

Antimicrobial agent	Year	Country and prevalence of resistance					
		
		DK	E	F	I	P	NL
Ampicillin	2002	-	0.0	20.4	-	13.0	-
	2003	0.0	10.0	5.0	11.1	-	25.7
	2004	-	2.0	-	-	-	6.3
Amoxicillin + Clavulanic. acid	2002	-	0.0	0.0	-	0.0	-
	2003	-	3.0	0.0	0.0	-	0.0
	2004	-	0.0	-	-	-	-
Ceftiofur	2002	-	-	0.0	-	-	-
	2003	0.0	-	0.0	-	-	0.0
	2004	-	-	-	-	-	0.0
Fluoroquinolones	2002	-	0.0	5.0	-	0.0	-
	2003	0.0	0.0	5.0	0.0	-	5.8
	2004	-	0.0	-	-	-	0.0
Florfenicol	2002	-	0.0	0.0	-	-	-
	2003	0.0	1.0	2.0	0.0	-	0.0
	2004	-	0.0	-	-	-	0.0
Tetracycline	2002	-	3.0	40.0	-	38.0	-
	2003	0.0	13.0	33.0	11.1	-	51.4
	2004	-	1.0	-	-	-	31.0
TMP + Sulphonamides	2002	-	1.0	16.6	-	-	-
	2003	0.0	4.0	16.0	0.0	-	8.6
	2004	-	0.0	-	-	-	0.0

DK – Denmark; E – England, F – France; I – Italy; NL – The Netherlands; P – Portugal.

**Table 4:** Occurrence of antimicrobial resistance among *Pasteurella multocida *isolated from cattle in different European countries

Antimicrobial agent	Year	Country and prevalence of resistance					
		
		DK	E	FIN	F	I	NL
Ampicillin	2002	0.0	1.0	-	5.3	-	-
	2003	6.25	0.6	0.0	2.0	0.0	2.2
	2004	-	1.0	-	-	-	0.0
Amoxicillin + Clavulanic acid	2002	-	2.0	-	0.0	-	-
	2003	-	0.0	-	0.0	0.0	-
	2004	-	0.0	-	-	-	-
Ceftiofur	2002	0.0	-	-	1.2	-	-
	2003	0.0	-	-	0.0	-	0.0
	2004	-	-	-	-	-	0.0
Fluoroquinolones	2002	0.0	0.0	-	4.7	-	-
	2003	0.0	0.0	0.0	0.0	0.0	0.0
	2004	-	0.0	-	-	-	6.3
Florfenicol	2002	0.0	2.0	-	0.0	-	-
	2003	0.0	0.6	-	0.0	0.0	0.0
	2004	-	0.0	-	-	-	0.0
Tetracycline	2002	12.0	6.0	-	10.7	-	-
	2003	0.0	4.0	0.0	12.0	24.0	20.0.
	2004	-	1.0	-	-	-	12.5
TMP + Sulphonamides	2002	8.0	3.0	-	7.0	-	-
	2003	0.0	1.0	0.0	3.0	4.0	2.2
	2004	-	<1.0	-	-	-	0.0

DK – Denmark; E – England, FIN – Finland; F – France; I – Italy; NL – The Netherlands.

**Table 5:** Occurrence of antimicrobial resistance among *Streptococcus spp*. from cases of bovine mastitis in different countries in Europe.

Antimicrobial agent	Year	Bacterial species, country and prevalence of resistance								
		
		*S. uberis*					*S. dysgalactiae*			
			
		E	F	I	NL	S	E	F	NL	S
		
Chloramphenicol	2002	-	-	-	-	0.0	-	-	-	0.0
	2003	-	-	7.0	-	-	-	-	-	-
	2004	-	-	-	-	-	-	-	-	-
		
Erythromycin	2002	8.0	22.2	-	21.4	0.0	3.0	14.8	14.0	0.0
	2003	8.0	17.0	22.0	19.3	-	5.0	17.0	12.8	-
	2004	8.0	-	-	0.0	-	2.0	-	6.7	-
		
Gentamicin	2002	-	52.7	-	-	-	-	11.7	-	-
	2003	-	-	52.0	-	-	-	-	-	-
	2004	-	-	-	-	-	-	-	-	-
		
Penicillin	2002	0.3	0.4	-	3.9	0.0	0.7	3.4	0.0	0.0
	2003	0.0	1.0	0.0	0.0	-	0.0	0.0	0.0	-
	2004	0.0	-	-	0.0	-	0.0	-	0.0	-
		
Tetracycline	2002	15.0	20.9	-	33.0	2.0	46.0	41.6	76.6	6.0
	2003	11.0	17.0	44.0	41.0	-	47.0	53.0	76.6	-
	2004	15.0	-	-	35.4	-	39.0	-	67.8	-
		
TMP + Sulphonamides	2002	-	0.8	-	8.7	0.0	-	6.6	0.0	0.0
	2003	-	0.0	-	1.2	-	-	4.0	0.0	-
	2004	-	-	-	0.0	-	-	-	0.0	-

E – England, F – France; I – Italy; NL – The Netherlands; S – Sweden.

**Table 6:** Occurrence of antimicrobial resistance among *Escherichia coli *isolated from cattle suffering from either diarrhoea or mastitis in different European countries.

Antimicrobial agent	Year	Country and prevalence of resistance									
		B	DK	E	F	I	LV	ES	NL	S	CH
		
Origin		Diarrhoea							Mastitis		
	
Ampicillin	2002	63.7	79.0	44.0	76.1	44.0	-	45.6	13.3	-	-
	2003	64.0	75.4	49.0	76.0	41.0	67.0	45.5	18.8	7.0	33.5
	2004	75.5	72.1	53.0	-	25.0	-	51.0	13.9	29.0	0.0
Amoxicillin + Clavulanic acid	2002	47.0	-	14.0	30.0	14.0	-	10.3	1.0	-	-
	2003	16.0	3.5	19.0	34.0	6.0	54.0	4.5	1.0	-	0.0
	2004	10.1	1.2	19.0	-	7.4	-	9.0	0.0	-	0.0
Apramycin	2002	10.8	0.0	3.0	17.2	-	-	3.7	-	-	-
	2003	10.0	3.6	3.0	11.0	-	-	5.2	-	0.0	-
	2004	8.0	0.0	3.0	-	-	-	6.0	-	-	-
Ceftiofur	2002	0.0	0.0	-	1.2	-	-	5.9^a^	0.0	-	-
	2003	2.0	0.0	-	0,7	-	0.0	2.6^a^	0.0	0.0	0.0
	2004	6.3	0.0	-	-	7.1	-	6.0^a^	0.0	0.0	-
Chloramphenicol	2002	54.9	13.0	-	65.4	30.0	-	29.4	-	-	-
	2003	48.0	14.1	-	58.0	21.0	-	33.8	-	2.0	18.0
	2004	52.7	22.8	-	-	-	-	31.0	-	5.0	13.3
Fluoroquinolones	2002	35.3	1.0	0.1	31.2	22.0	-	11.8	0.0	-	-
	2003	26.0	1.8	0.04	20.0	15.0	0.0	12.3	0.0	1.0	6.0
	2004	32.1	7.6	0.0	-	11.5	-	20.0	1.0	10.0	0.0
Florfenicol	2002	31.4	1.0	-	12.6	-	-	8.1	-	-	-
	2003	26.0	3.5	-	12.0	-	-	9.7	-	0.0	-
	2004	21.5	5.1	-	-	-	-	8.0	-	0.0	-
Gentamicin	2002	53.3	1.0	-	21.6	12.0	-	14.0	0.0	-	-
	2003	16.0	3.6	-	20.0	1.0	29.0	19.5	0.0	0.0	6.0
	2004	7.6	7.6	-	-	7.1	-	25.0	0.0	0.0	6.7
Nalidixic acid	2002	42.2	19.0	-	35.0	30.0	-	21.3	1.0	-	-
	2003	38.0	14.1	-	36.0	21.0	56.0	18.8	-	3.0	-
	2004	54.0	29.1	-	-	21.4	-	31.0	-	14.0	-
Neomycin	2002	46.0	9.0	26.0	42.5	-	-	25.0	7.6	-	-
	2003	26.0	15.8	27.0	44.0	-	86.0	25.0	6.9	1.0	18.0
	2004	17.7	45.6	30.0	-	-	-	26.0	5.0	7.0	-
Streptomycin	2002	-	70.0	-	78.6	24.0	-	52.2	15.2	-	-
	2003	-	68.4	-	78.0	54.0	69.0	54.8	20.8	11.0	-
	2004	-	87.3	-	-	25.0	-	55.0	15.8	48.0	-
Sulphonamides	2002	-	74.0	-	80.6	46.0	-	45.6	-	-	-
	2003	-	65.0	-	69.0	47.0	100.0	52.9	-	8.0	-
	2004	-	94.9	-	-	25.0	-	51.0	-	41.0	-
Tetracycline	2002	58.8	84.0	48.0	79.5	56.0	-	49.3	14.3	-	-
	2003	64.0	75.5	52.0	76.0	52.0	71.0	56.5	20.8	5.0	24.0
	2004	76.4	84.8	57.0	-	28.6	-	59.0	14.9	37.0	20.0
Trimethoprim	2002	-	54.0	-	52.4	-	-	33,1	-	-	-
	2003	-	52.6	-	39.0	-	0.0	41.9	-	3.0	-
	2004	-	70.9	-	-	-	-	41.0	-	10.0	-
TMP + Sulphonamide	2002	56.9	-	23.0	39.5	26.0	-	-	11.4	-	-
	2003	44.0	-	27.0	35.0	24.0	0.0	-	10.9	-	12.5
	2004	54.0	-	28.0	-	8.7	-	-	8.9	-	-

a: Cefotaxime

B – Belgium; DK – Denmark; E – England; F – France; I – Italy; LV – Latvia; NL – The Netherlands; ES – Spain; S – Sweden; CH – Switzerland.

### Quality control

Invitation was annually announced through the network by email or facsimile to all ARBAO members to participate in self-evaluating proficiency tests (EQAS external quality assurance system) for antimicrobial susceptibility testing. The tests were conducted each year to test if the current methodologies were accurate, adequate and reliable [3,4]. In addition, the EQAS served as a tool to point out the laboratories from where annual data were reliable. The goal was to have all laboratories to perform antimicrobial susceptibility testing with a maximum of 10% total deviations (minor, major, or very major deviations) and a maximum of 5% critical deviations (major or very major deviations).

Eight strains of each species (*Staphylococcus aureus, Mannheimia haemolytica, Pasteurella multocida*, *Streptococcus dysgalactiae, Streptococcus uberis *and *Escherichia coli) *were selected for each EQAS iteration. Strains were obtained from the isolate collection of the National Food Institute, Technical University of Denmark (DTU Food). All strains were included in only one EQAS iteration. The strains were inoculated to agar stab cultures for shipping to participating laboratories. Participating laboratories also received a lyophilised reference strain as a quality control strain for susceptibility testing (*E. coli *ATCC 25922; *Campylobacter jejuni *ATCC 33560; *Enterococcus faecalis *ATCC 29212, *S. aureus *ATCC 25923, and *S. aureus *ATCC 29213) in each EQAS shipment.

Participating laboratories were instructed to follow the enclosed testing instructions; subculture the test strains, and propagates the quality control strains prior to performing the susceptibility method that was routinely used by their laboratory. In addition, laboratories were advised to maintain the quality control strain for future proficiency tests. After completion of the susceptibility testing of the test strains and the quality control strain, the participating laboratories were instructed to record the obtained results, using MIC values or zone-diameter in millimetres, and categorize each of the tested strains as either "resistant" (R), "intermediate" (I) or "susceptible" (S) against each tested antimicrobial agent using the breakpoints routinely used in their laboratory. They were then asked to record the information on the participating laboratory record sheet.

After submitting results, participating laboratories received an individual report. The individual reports for the participating laboratories reported all deviations from the expected values and suggestions of how to either solve or investigate the problem. For the quality control strains, deviations were defined as values that exceeded the quality control range of the strain. Deviations of the antimicrobial susceptibility results were categorised as minor, major or very major. A minor deviation was defined as an intermediate strain that was classified as susceptible or resistant or vice versa (i.e. I ↔ S or I ↔ R). A major deviation was defined as a susceptible strain that was classified as resistant (i.e. S → R). A very major deviation was defined as a resistant strain that was classified as susceptible (i.e. R → S).

The overall performance was between 85%–100% correctly categorized isolates. In general, the concordance between the expected results and the participants' results for *Enterobacteriaceae *was highest. Data from laboratories performing unsatisfactory in the EQAS (performance below 90% correctly categorized results) were not included in the present report. The summary results for the bacterial species included in the EQAS are considered to be comparable between countries, but there is a potential bias due to differences in sampling and microbiological methods used for isolation in the different countries. The EQAS results for streptococci and staphylococci were less favourable that for *Enterobacterriaceae*.

### Testing methods

Each participating laboratory reported annually which methods were used for antimicrobial susceptibility testing. Laboratories from England (and Wales), France, Italy, Latvia and Portugal used disc diffusion tests, whereas Denmark, the Netherlands, Norway, Sweden and Switzerland used the broth micro dilution method by which the minimal inhibition concentration (MIC) is determined. Spain and Finland both used MIC determination as well as disc diffusion, whereas Belgium used tablet diffusion. The manufactures of discs and microtitre plates for broth micro dilution differed between countries.

All countries have reported whether they to some extend or in all aspects followed the standards of the Clinical and Laboratory Standards Institute (CLSI), M31-A2, M7-A6 or M2-A8 [5] to determine zone sizes and MIC values. England (and Wales), France, the Netherlands, Norway, Sweden and Switzerland reported that they did not completely use CLSI breakpoints as their means of determining the antimicrobial susceptibility levels (referring to the CLSI breakpoints listed in the fifteenth international supplement M100-S15). The antimicrobials tested against *S. aureus, M. haemolytica, P. multocida*, *S. dysgalactiae, S. uberis *and *E. coli *are given in Tables 2, 3, 4, 5, 6.

## Results

Susceptibility data were obtained from 25,241 bacterial isolates isolated in 13 European countries over a three year period, 2002–2004 (Table 1). Not all countries were equal participants in that some countries submitted data on a single bacterial species, e.g. Belgium, Finland, Latvia, Portugal and Norway, whereas other countries provided data for multiple bacterial species.

### Staphylococcus aureus

Susceptibility data for *S. aureus *from bovine mastitis were obtained from ten of the 13 participating countries. In total between 691–1,321 isolates were tested depending on the year and number of countries submitting date for that year (Table 1). In general, a relatively low frequency of resistance was observed in all countries over the years (Table 2). However, a higher level, e.g., > 10%, resistance was noted to penicillin in almost all countries with the exception of France, Norway and Sweden.

### Mannheimia haemolytica

Susceptibility data for *M. haemolytica *from infections in cattle were obtained from a total of six countries (Table 3). The six countries tested between 117–221 isolates. In general, the level of resistance was relatively low with the exception of tetracycline and ampicillin in all countries over the years (Table 3). Resistance to ceftiofur was not detected among the 529 isolates tested.

### Pasteurella multocida

Susceptibility data for *P. multocida *from infections in cattle were obtained from a total of six countries. Results from a total of 194–504 tested isolates were submitted (Table 1). In general, a relatively low frequency of resistance was observed in all countries over the years (Table 4).

### Streptococcus uberis

Susceptibility data for *S. uberis *from mastitis in cattle were obtained from five countries (Table 1). The number of isolates tested was 984–1,687. In general, a relatively moderate frequency of resistance was observed in all countries over the years (Table 5). Major differences were observed between countries and some variation also over years.

### Streptococcus dysgalactiae

Susceptibility data for 255–521 *S. dysgalactiae *isolates from mastitis in cattle were obtained from the same countries as for *S. uberis *(Table 1). In general, there was an increased frequency of resistance observed in all countries over the years. Most noticeable was a very high level of resistance against tetracycline (Table 5). Major differences were observed between countries and some variation also over years.

### Escherichia coli

Susceptibility data for *E. coli *from diarrhoea in calves and mastitis in cattle and surveillance programmes were obtained from ten countries (Table 6), which submitted susceptibility data for 3 490–5,921 isolates. A high prevalence of resistance was observed in this pathogen compared to the other species investigated (Table 6). Major variations between countries and antimicrobial agents were apparent.

## Discussion

The data presented in this report originate from samples submitted to diagnostic laboratories in different countries. It was generally impossible to retrieve data on the specific age of the animals and additionally, the specific microbiological methods used to isolate and identify the organisms remained unreported. All participating laboratories are, however, appointed national reference laboratories for antimicrobial resistance.

Information on antimicrobial usage prior to collection of the samples could have indicated why resistance in some countries is higher than in others, and also, it could have indicated a reason for the difference seen between antimicrobials. As information on these key determinants is not available, comparisons and conclusions have to be done with care. Nevertheless, the study provides interesting data on the prevalence of antimicrobial resistance in bacterial pathogens in different European countries.

Although there was a prior agreement between the participating laboratories on a list of antimicrobials of relevance for each bacterial species, most laboratories provided data for different panels of antimicrobial agents. The same heterogeneity was observed in the breakpoints applied. This demonstrates an important problem in performing international monitoring based on data produced by routine diagnostic laboratories using different panels, methods, equipment etc. [6,7]. We are aware that publishing data from multiple laboratories can be very problematic as there might be variations in methods used, interpretative criteria, etc. In the ideal world all laboratories would use the same methods and we could believe that data were directly comparable. However, since this unfortunately not is the case and there actually are major differences in the methods used we decided to ensure the comparability of the data as means of gaining reliable by conducting an external quality control system and only include data from laboratories and pathogens where the cut-off was met.

### Staphylococcus aureus

In a previous survey of isolates from nine European countries and USA, major variations in the occurrence of resistance between countries were reported [8]. This emphasises that treatment strategies for bovine mastitis caused by *S. aureus *in the European countries have to be based on local knowledge and available resistance data. In the present study, it seems that the level of resistance in France has decreased between the years 2002 and 2003; for oxacillin (8.3% to 1%), for sulphonamide (59.2% to 2%) and for trimethoprim (11.1% to 0%). It was not possible to detect the same trend in the year 2004 due to the absence of submitted data. Similar trends were only observed in one other country (Switzerland), where a considerable decrease of gentamicin resistance from 30% to 2.5% was observed.

Of major concern is the level of resistance to oxacillin and 3^rd ^generation cephalosporins (i.e. ceftiofur) in *S. aureus*. The prevalence of oxacillin resistance in Spain (3.7%) and France (8.3%) and the resistance towards cephalosporins in Spain (0.9% in 2004) and France (4.2% in 2002; 1% in 2003) indicate the presence of methicillin resistant *S. aureus *(MRSA) in these two countries. Most of the Spanish isolates were not multi resistant which usually a feature of MRSA isolated from humans is. Furthermore, the presence of the *mecA-*gene could not be confirmed since the strains were not stored.

For France the percentages of resistance observed by oxacillin resistant *S. aureus *isolates must be interpreted with a great deal of precaution. Data on bovine pathogens were collected through a multi centre study (see Table 1). The results could not be verified because some of the laboratories involved in the network did not store the strains. From 2003, all the strains collected in France were investigated specifically for methicillin resistance and a decrease was observed. MRSA are not commonly seen in bovine mastitis but this has recently been reported in Korea [9] and Hungary [10]. MRSA also seems to be emerging within other livestock species [11,12].

### Mannheimia haemolytica

The decrease in resistance levels to antimicrobials in isolates from the Netherlands to ampicillin, fluoroquinolone, tetracycline, trimethoprim – sulphonamide may partly be explained by sampling bias (sampling of different age groups between years). The same tendency was observed in both England (and Wales) and France to some of the antimicrobials. In France the level of resistance to ampicillin and tetracycline decreased from 2002 to 2003 whereas a decrease in England (and Wales) was observed for ampicillin, amoxicillin – clavulanic acid, tetracycline and trimethoprim – sulphonamide in the period 2003 to 2004. Resistance to fluoroquinolones was detected in France with 5% (2002) and in the Netherlands (enrofloxacin) with 5.8% (2003). Similar results have previously been reported from France [13] and the Netherlands [14], whereas a similar or more frequent occurrence of resistance has been observed in USA [15,16].

### Pasteurella multocida

Antimicrobial resistance in *P. multocida *seems to be higher in the Netherlands compared to the other countries (e.g. 20% to tetracycline in 2002) though the amount of resistance shows a tendency to decrease over the years for ampicillin, tetracycline and trimethoprim – sulphonamide (Table 4). The same trend was observed in England (and Wales), France and Denmark to tetracycline and trimethoprim – sulphonamide. It should be noted, that in 2002 in France 1.2% of the isolates were resistant to ceftiofur and 4.7% to fluoroquinolones. Resistance to enrofloxacin in the Netherlands was 6.3% in 2004.

### Streptococcus uberis

Resistance to trimethoprim – sulphonamide and penicillin seems to be low compared to the levels of resistance for other antimicrobials tested. The percentages of resistance to trimethoprim + sulphonamide (8.7%) and penicillin (3.9%) were the highest values observed in the Netherlands. A relatively high level of resistance could be detected in countries, which submitted data for tetracycline and erythromycin as well as gentamicin (Table 5). Similar data have been reported from USA [17,18] and France [19]. Thus, bovine mastitis caused by streptococci can be treated with penicillins, whereas the use of macrolides and tetracyclines probably should be avoided on the basis of the resistance data generated by this study.

### Streptococcus dysgalactiae

Resistance to tetracycline and erythromycin was high compared to the other antimicrobials tested. In the Netherlands 67.8% of the isolates were resistant to tetracycline in 2004 and 17% of the isolates from France were resistant to erythromycin in 2003. The isolates from Sweden were susceptible to all antimicrobials tested, except for tetracycline, where the level was 6% (Table 5).

### Escherichia coli

The percentage of resistant isolates increased in some countries, whereas it decreased in others. For instance in France and Italy the resistance levels show a tendency to decrease for most of antimicrobials investigated between the years 2002–2004. In Spain and Denmark the levels seem to increase between the years 2003 and 2004. In general, a relatively high frequency of resistance was observed in almost all countries over the years, with the exception of Sweden and the Netherlands the frequency was more moderate. Resistance to ceftiofur, representing the 3^rd ^generation cephalosporins, was observed in France (2002, 1.2%; 2003, 0.7%), Spain (2002, 5.9%; 2003, 2.6%; 2004, 6%), Italy (2004, 7.1%) and arising in Belgium (2003, 2%; 2004, 6.3%). The frequent occurrence of resistance to more conventional antimicrobials in most of these countries makes cephalosporins a group of drugs that can be used without prior susceptibility testing. Greater than 10% fluoroquinolone resistance was observed in Sweden (enrofloxacin), France, Spain, Belgium, Germany and Italy. Higher rates of resistance towards newer antimicrobials (e.g. fluoroquinolones, aminoglycosides) may be due to the attitudes and practice among European countries in prescribing and administering drugs for therapy of enteric and respiratory tract diseases [20].

The seemingly emerging occurrence of resistance to the important antimicrobial agents; cephalosporins and fluoroquinolones in Belgium, France, Italy and Spain is worrying. These four countries had in general the highest frequency of resistance to most antimicrobial agents, potentially making treatment difficult.

## Conclusion

A frequent occurrence of resistance to several antimicrobial agents was observed and major differences between countries are apparent. This may reflect differences in the antimicrobial use between countries and veterinarians.

This study supports the premise that the treatment of infected animals has to be based on local knowledge and the observed resistance patterns.

Some limitations in the study design were apparent. Because of this precautions should be taken when comparing the summary data. Furthermore, differences in antimicrobial resistance testing and to some extend differences in breakpoint used may also complicate the comparison between the countries. The use of an external quality assurance system did, however, reveal that the influence of the differences and lack of standardisation might be minor.

## Competing interests

The authors declare that they have no competing interests.

## Authors' contributions

RSH provided data, discussed the results gained, drafted, and revised the manuscript. DJM, AS, CT, DM, PB, AF, AU, AA, MM, CG, KS, CB, AM, DW, and MS provided data, discussed the results gained, and participated in revising the manuscript. FMA provided data, discussed the results gained, assisting drafting and revision of the manuscript. All authors read and approved the final manuscript.
